# Mapping the oxidative stress metabolome in neurology by gas chromatography–mass spectrometry: a systematic review on signature-driven diagnosis and disease monitoring

**DOI:** 10.1016/j.redox.2025.103925

**Published:** 2025-11-10

**Authors:** Cristina Lorca, Aida Serra, Xavier Gallart-Palau

**Affiliations:** a Biomedical Research Institute of Lleida Dr. Pifarré Foundation (IRBLLEIDA), +Pec Proteomics Research Group (+PPRG), Neuroscience Area, University Hospital Arnau de Vilanova-ICS (HUAV), 80 Av. Rovira Roure, Lleida, 25198, Spain; b Department of Medical Basic Sciences (CMB), University of Lleida (UdL), +Pec Proteomics Research Group (+PPRG), Lleida, 25198, Spain; c Departament d’Infermeria i Fisiotèrapia, Universitat de Lleida (UdL), 25198, Lleida, Spain

**Keywords:** Oxidative stress, Neurology, Biomarkers, Metabolomics, Gas chromatography–mass spectrometry

## Abstract

Oxidative stress is a pivotal factor in the pathogenesis of neurological conditions; however, its clinical assessment is constrained by the ephemeral nature of reactive species and the variability of analytical methodologies. Gas chromatography coupled with mass spectrometry (GC-MS) provides high sensitivity, molecular specificity, and well-established libraries, positioning it as a promising translational platform for the mapping of oxidative-stress–related metabolites in neurological disorders. We conducted a PRISMA-guided, PROSPERO-registered systematic review of human studies employing GC-MS to quantify oxidative-stress–linked metabolites in central nervous system disorders (January 2014–January 2025). Two independent reviewers screened records from PubMed and Scopus, extracted study and assay characteristics, and evaluated bias using design-appropriate tools. Twenty-four studies met the inclusion criteria, encompassing neurodegenerative, injury-related, infectious, and psychiatric conditions. Blood was the most frequently utilized matrix (14/24), with neurodegenerative diseases being the most represented (10/24). Across these studies, 70 metabolites were identified as significantly altered compared with controls. Consistent findings were associated with lipid peroxidation (e.g., isoprostanes, neuroprostanes, oxysterols), glutathione cycling and amino acid redox pathways (e.g., cystine, pyroglutamate), energy metabolism (e.g. TCA intermediates, lactate, pyruvate), purine turnover and oxidative DNA damage markers, as well as sugars/polyols implicating the pentose-phosphate and polyol pathways. These results underscore oxidative stress as a convergent mechanism linking neuroinflammation, mitochondrial dysfunction, and membrane damage across central nervous system disorders, and highlight GC–MS–derived metabolite panels as emerging candidates for diagnosis and monitoring. Standardized, multi-matrix protocols, untargeted discovery, targeted validation, and longitudinal cohorts are now required to define robust stress-related metabolomic signatures and advance clinical translation in neurology.

## 1 Introduction

Oxidative stress is defined as an imbalance between oxidants and the capacity of biological systems to neutralize them through antioxidant mechanisms, resulting in potential molecular and cellular damage [1]. While excessive oxidative stress is deleterious, it is important to recognize that, at low to moderate concentrations, oxidant molecules such as reactive oxygen species (ROS) or reactive nitrogen species (RNS) play essential physiological roles in processes such as cellular signaling, immune responses, regulation of gene expression and homeostasis [2]. Moreover, transient and manageable increases in oxidative stress, as those happening during physical exercise or exposure to mild stressors, can generate adaptive responses that enhance the organism's resilience to future stress. This phenomenon, known as hormesis, has extensive benefits for the organism, including the upregulation of antioxidant defenses, DNA repair mechanisms, and mitochondrial biogenesis, contributing to improved cellular and systemic function [3].

To maintain a proper redox balance, human cells possess a complex network of antioxidant systems. These include enzymatic antioxidants, such as superoxide dismutase (SOD), which catalyzes the dismutation of superoxide into hydrogen peroxide; catalase (CAT), which breaks down hydrogen peroxide into water and oxygen; and glutathione peroxidase (GPx), which reduces hydrogen peroxide and lipid peroxides using glutathione as a cofactor [4]. In parallel, non-enzymatic antioxidants, like vitamins C and E, glutathione, flavonoids, and carotenoids, neutralize oxidant molecules directly and support enzymatic systems indirectly through redox cycling and regeneration of antioxidant enzyme cofactors [4]. When the generation of oxidants overwhelms these defenses, excessive levels of oxidant molecules can lead to nonspecific oxidation and widespread molecular damage. The damage to molecules such as lipids, proteins, and DNA can cause loss of membrane integrity, impaired enzymatic activities, and genomic instability, compromising cellular viability and function [2]. Chronically elevated oxidative stress can further initiate or exacerbate inflammation, disrupt redox-sensitive signaling pathways, and contribute to the pathophysiology of numerous chronic diseases [5]. At the tissue and systemic levels, sustained oxidative stress has been implicated in endothelial dysfunction, neurodegeneration, hepatic injury, and cardiovascular disease, among other conditions [6]. The central role of oxidative stress in these disorders underlines the importance of understanding and monitoring redox status in both research and clinical contexts.

It is also relevant to recognize that an imbalance in the opposite direction, toward excessive reduction, is also pathological. This situation, known as reductive stress, is characterized by the excessive accumulation of reducing molecules, such as NADH, NADPH, and reduced glutathione [7]. Although significantly less common, an overly reduced cellular environment can also lead to cellular damage by impairing ROS-mediated signaling, which is essential for physiological processes such as cell proliferation, differentiation, and apoptosis [8]. Furthermore, reductive stress has been associated with protein misfolding, endoplasmic reticulum stress, lysosomal and mitochondrial dysfunction, and the development of certain pathologies, such as hereditary cardiomyopathy and metabolic disorders [7]. Thus, maintaining a proper redox balance, neither excessively oxidative nor reductive, is fundamental to cellular homeostasis and organismal health.

The exposure and use of oxygen, essential for aerobic life, naturally leads to the generation of oxidant species and gradual accumulation of oxidative damage over time, a process that has long been associated with aging [9,10]. Mitochondria, immune responses, and cellular metabolism are the main internal sources of oxidant molecules [10]. Mitochondria, in particular, contribute to oxidative stress through electron leakage at complexes I and III of the electron transport chain, resulting in superoxide generation [11]. Additionally, environmental exposures and modern human lifestyle factors have significantly increased the oxidative burden in humans. These include chronic psychological stress, increased exposure to air pollutants, industrial chemicals, xenobiotics, and radiation—all of which contribute to sustained ROS production and redox imbalance [12].

A common mechanistic pathway involves the activation of immune cells in chronic inflammation, leading to the release of ROS and RNS as part of the host defense mechanism [13]. Likewise, mitochondrial dysfunction, whether due to aging, environmental stress, or genetic predisposition, can lead to electron leakage and an overproduction of ROS, further compounding oxidative damage and impairing energy metabolism [11]. These factors contribute to depleting the antioxidant defenses, promote cellular senescence, and drive progressive organismal dysfunction.

In this context, it is not surprising that oxidative stress has been strongly implicated in a wide spectrum of human pathologies, including cardiovascular, metabolic and neurodegenerative disorders [6]. The nervous system, due to its high metabolic demand, lipid-rich composition, and relatively low antioxidant capacity, is highly vulnerable to oxidative damage [14]. As such, oxidative stress plays a central role in the pathogenesis of numerous neurodegenerative diseases, including Alzheimer's disease, Parkinson's disease, amyotrophic lateral sclerosis, and multiple sclerosis [15]. Furthermore, oxidative stress is also involved in acute neurological injuries such as ischemic stroke and traumatic brain injury, where excessive ROS production during reperfusion or injury response can exacerbate neuronal death and worsen functional outcomes [16]. Oxidative molecular damage can directly induce neuronal death, neuroinflammation, and progressive neurological dysfunction [15].

Due to their highly reactive and short-lived nature, measuring the presence of oxidant molecules directly remains technically challenging. Fluorescent and chemiluminescent probes, such as 2′,7′-dichlorodihydrofluorescein diacetate (DCFH-DA), are commonly used for the indirect detection of ROS in biological systems. Upon oxidation by ROS, DCFH-DA is converted into the fluorescent compound DCF, providing a measure of general oxidative activity, though it lacks specificity for individual ROS types [17]. Another direct detection method is electron spin resonance (ESR) spectroscopy, which allows for the measurement of unpaired electrons in free radicals. When used in combination with spin-trapping agents, ESR can provide highly specific information about individual radical species [17,18].

Oxidative stress is usually assessed by measuring end-products of oxidative damage, which are more stable. Reliable biomarkers include lipid, protein, and DNA oxidation products. Lipid peroxidation markers include malondialdehyde, 4-hydroxynonenal or F2-isoprostanes (F2-IsoPs). Examples of protein oxidation markers are protein carbonyls, advanced oxidation protein products (AOPPs), or 3-nitrotyrosine. Molecules like 8-hydroxy-2′-deoxyguanosine (8-OHdG) or formamidopyrimidines serve as markers of DNA oxidation [17].

Oxidative stress status can also be inferred from the evaluation of antioxidant systems. One of the most informative indicators is the glutathione redox ratio (GSH/GSSG), which reflects the balance between reduced and oxidized glutathione and serves as an indicator of oxidative stress severity [19]. Antioxidant enzymatic activity also serves as an indirect but functional insight into oxidative stress levels. It can be determined by measuring the activity of enzymes like SOD, which catalyze the dismutation of superoxide radicals, and CAT and GPx, involved in hydrogen peroxide detoxification [4]. Other relevant biomarkers include oxidized low-density lipoprotein (oxLDL), which reflects lipid peroxidation in the vascular system and is linked to atherosclerosis [20], and 3-nitrotyrosine, which indicates peroxynitrite-related oxidative protein modification [21]. Additionally, certain circulating metabolites such as homocysteine or uric acid can also serve as indirect indicators of oxidative stress and redox imbalance [22].

The method selected for measuring oxidative stress will depend on several factors, including the type of biological sample available, the specific biomarkers of interest, and the required analytical sensitivity and specificity. Given the complexity and redundancy of redox-related pathways, it is generally recommended to assess multiple biomarkers representing different molecular targets—such as lipids, proteins, and nucleic acids—to obtain a comprehensive and reliable assessment of oxidative stress status [17].

Among available analytical technologies, mass spectrometry (MS) stands out as one of the most powerful tools for the identification and quantification of small-molecule biomarkers, including those associated with oxidative stress. MS provides high sensitivity, selectivity, and molecular specificity. MS is typically preceded by a separation step to resolve individual components in complex biological samples. Separation techniques are usually selected based on the physicochemical properties of the target compounds, and include: liquid chromatography (LC), gas chromatography (GC), capillary electrophoresis (CE), thin-layer chromatography (TLC), and high-performance liquid chromatography (HPLC) [23]. Once separated, individual compounds are ionized, or converted into small, charged particles, using techniques such as electrospray ionization (ESI) or matrix-assisted laser desorption/ionization (MALDI) for LC-MS, or electron ionization (EI) for GC-MS. Once ionized, molecules are introduced into a mass analyzer, which measures the mass-to-charge ratio (*m*/*z*) of the ions. Examples of the most widely used mass analyzers are quadrupole time-of-flight (QTOF), orbitrap, and Fourier transform ion cyclotron resonance (FT-ICR). The acquired spectral data are then processed using computational tools that perform peak detection, feature alignment across samples, and fragmentation pattern extraction. These features are then compared against spectral libraries and compound databases to identify potential matches [23].

Specifically, GC-MS is widely recognized for its exceptional sensitivity, accuracy, and reproducibility, primarily due to the superior separation efficiency of GC [24]. In GC-MS, the sample is first vaporized and introduced into a gas chromatographic column, where analytes are separated based on differences in volatility and affinity for the stationary phase. To undergo GC separation, the analytes must be volatile and thermally stable. Therefore, non-volatile or thermolabile molecules—such as amino acids, sugars, fatty acids, organic acids, and nucleotides—typically require chemical derivatization prior to analysis. Derivatization enhances their volatility, thermal stability, and chromatographic performance by reducing polarity and increasing hydrophobicity. Common derivatization strategies include silylation (e.g., using MSTFA or BSTFA) for hydroxyl, carboxyl, and amino groups; methylation or acetylation for acids or amines; or oximation to stabilize carbonyl-containing compounds [25].

A subset of biological molecules can be directly analyzed by GC-MS without the need for derivatization [26]. Among these, volatile organic compounds (VOCs), which are low-molecular-weight, carbon-based chemicals with high vapor pressure at room temperature, are of particular interest. VOCs are present in samples such as breath, urine, sweat, and blood [27]. They originate from both endogenous metabolic processes and exogenous sources, such as diet, the microbiota, environmental exposures, and lifestyle habits. Because their production and release are influenced by physiological and pathological states, VOCs are increasingly recognized as non-invasive biomarkers for disease diagnosis, monitoring, and prognosis. For example, altered VOC profiles have been reported in the breath of patients with lung cancer, type 2 diabetes, and inflammatory conditions, among others [28]. Notably, many VOCs are also directly linked to oxidative stress, as several are byproducts of lipid peroxidation [26], a process in which ROS oxidize polyunsaturated fatty acids in cell membranes. Key oxidative stress-related VOCs include short-chain alkanes (generated from omega-3 and omega-6 lipid peroxidation), aldehydes (arising from hydroperoxide breakdown), and ketones and alcohols (associated with metabolic imbalance and redox shifts) [29]. These compounds are being actively studied as real-time, non-invasive indicators of oxidative damage in diseases such as asthma, chronic obstructive pulmonary disease (COPD), cardiovascular disease, and neurodegenerative disorders [26,28]. In addition to VOCs, other molecules directly detectable through GC-MS include short-chain fatty acids (e.g. acetic, propionic, and butyric acid) [30], environmental and exogenous substances (e.g. nicotine, caffeine, solvents and certain drugs), and a few low-polarity steroids and sterols (e.g. cholesterol and testosterone), which can be measured directly under optimized conditions [31]. While derivatization expands the range of compounds suitable for GC-MS analysis, a subset of volatile, low-polarity, and thermally stable molecules can be analyzed directly, facilitating rapid, robust, and non-invasive assessments of metabolic and oxidative states.

As previously discussed, due to the high brain sensitivity to oxidative damage [32], excessive oxidative stress in the central nervous system (CNS) has been strongly implicated in a variety of neurological conditions. Historically, the CNS was viewed as a relatively isolated compartment, protected from the rest of the body by the blood-brain barrier (BBB). However, mounting evidence indicates that this highly specialized interface, while crucial to preserving the delicate neural microenvironment, is not an infallible gateway [33]. In addition to small molecules such as essential nutrients and oxygen, larger molecules, including bacterial proteins and other microbial components, have been detected within brain tissue. It is now widely recognized that the BBB is dynamic and context-sensitive, and can become functionally compromised under states of inflammation, infection, trauma, or neurodegeneration [33].

This bidirectional communication between the brain and peripheral systems supports the notion that systemic biological fluids can reflect CNS physiological and pathological states, particularly when analyzed using high-sensitivity, high-resolution analytical platforms. The increasing availability of high precision and high-throughput analytical technologies, such as MS, which enables the untargeted or targeted detection of thousands of molecular features simultaneously, provides an unprecedented systems-level view of human biology. These advances are transforming the biomedical paradigm from a reductionist, organ-based model to one rooted in systems biology, where diseases are viewed as complex network disruptions. Despite their inherent complexity, omics-based approaches, including metabolomics, proteomics, and lipidomics, are enabling deep mechanistic insights into disease processes [23]. By detecting subtle molecular perturbations prior to the onset of clinical symptoms, omics-based tools provide a foundation for predictive and preventive medicine, supporting earlier diagnosis, patient stratification, and individualized therapeutic interventions.

This systematic review focuses on a small but highly relevant piece of the puzzle: the application of GC-MS for the detection of oxidative stress biomarkers in human biological samples, with particular focus on their relevance to neurological diseases. GC-MS, a highly sensitive and selective platform, is particularly suited for analyzing volatile and semi-volatile compounds, including those produced by lipid peroxidation and other redox-related pathways, in accessible peripheral matrices [23,24]. The study of oxidative stress-related biomarkers in peripheral fluids offers a non-invasive window into brain health and represents a valuable diagnostic and prognostic tool. Although focused on a specific analytical strategy, this approach contributes meaningfully to the broader landscape of precision medicine, offering novel opportunities for non-invasive and personalized management of neurological diseases.

## 2 Methods

### 2.1 Study design

This systematic review was conducted according to PRISMA (Preferred Reporting Items for Systematic Reviews and Meta-Analyses) guidelines [34], in two different databases: PubMed and Scopus, by two independent reviewers. The search protocol was prospectively registered in PROSPERO. The full search strategy can be accessed through this link:

https://www.crd.york.ac.uk/PROSPEROFILES/b2e1bb172d51d4a9c1b0016d2b00b6e6.pdf.

### 2.2 Eligibility criteria

All studies reviewed used GC-MS technology to measure compounds related to oxidative stress in CNS diseases in different types of human samples and were published from January 2014 onward. Non-primary research articles, as well as studies conducted *in vitro* or in animal models, were excluded. Only studies including properly matched healthy controls and demonstrating a low or moderate risk of bias were included. The details on study design, population characteristics, and control selection for each study are provided in Table 1 of the Supplementary materials.

**Table 1:** **PRISMA flow diagram for the systematic search strategy,** following the directives of PRISMA 2020 (61). The search strategy followed in this systematic review was divided into three different steps: (A) Identification: PubMed and Scopus databases were screened, yielding 168 and 237 articles, respectively. (B) Screening: duplicates were excluded, and the remaining articles were preselected by title and abstract, followed by full-text assessment according to inclusion criteria^a^. (C) Inclusion: a total of 24 articles met eligibility requirements and were finally included. Additional references cited in the Introduction and Discussion are not shown in this flowchart.

Databases	PubMed	Scopus
**Identification**	168	237
	72 duplicates removed → 333 articles	
**Screening**	309 articles excluded	
**Inclusion**	24 articles included	

a Inclusion criteria: publication date January 2014–January 2025; analysis by gas chromatography–mass spectrometry of molecules related to oxidative stress in human samples from individuals with central nervous system disease compared with healthy controls; sample size n > 10; article available in English language.

### 2.3 Data extraction

The search strategy was performed by two independent reviewers. First, titles and abstracts were screened against predefined inclusion and exclusion criteria. Afterwards, the full text of potentially eligible studies was assessed before final inclusion. Any disagreements between the reviewers were resolved by discussion or by involving a third reviewer. Key information extracted included study characteristics (e.g., authors, year of publication, study design), participant details (e.g., neurological condition, sample size, demographics), associations with oxidative stress, analytical methods for detection and quantification, any reported diagnostic utility, and any measures of study quality or bias for each study.

### 2.4 Risk of bias assessment

Study quality was evaluated according to the Newcastle-Ottawa-Scale for cohort studies, while the QUADAS-2 and Cochrane Risk of Bias (RoB 2.0) tools were employed for diagnostic accuracy studies and randomized control trials, respectively.

## 3 Results

### 3.1 Study characteristics

A total of 405 articles were identified through the systematic search strategy, of which 333 remained after duplicate removal. Of these, 24 articles met the eligibility criteria and were included. The PRISMA flow diagram illustrating the selection process is shown in Table 1. Neurodegenerative diseases were the most studied category, accounting for 10 out of 24 articles. Blood was the most frequently analyzed sample, with 14 studies, yielding 44 metabolites associated with oxidative stress that showed significant differences in concentration across neurological diseases compared with healthy controls. Table 2 summarizes the number of articles and metabolites identified by disease group and sample type.

**Table 2:** Number of articles and metabolites identified according to disease type and type of sample analyzed.

Sample/Disease type	Neurodegenerative	Psychiatric-psychological	Injury	Infection	Other	All diseases
**Articles identified**						
Blood	7	2	3	1	1	14
Urine	1	1	0	0	1	3
Cerebrospinal fluid	1	0	1	1	0	3
Other	1	2	0	0	1	4
All samples	10	5	4	2	3	24
**Metabolites identified**						
Blood	29	6	14	3	5	44
Urine	4	7	0	0	6	16
Cerebrospinal fluid	3	0	2	2	0	7
Other	3	16	0	0	4	22
All samples	35	26	15	3	14	70

### 3.2 Metabolites identified

**Fig. 1:**
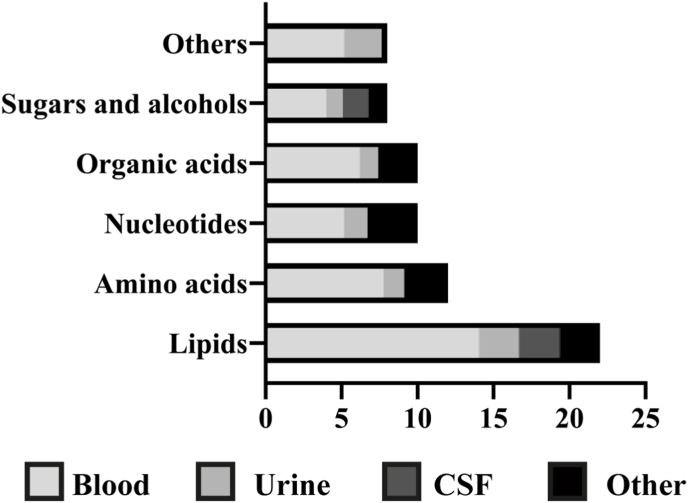
Number of oxidative stress-related metabolites showing significant differences between patients with CNS diseases and healthy controls, classified by molecular class and sample type.

The systematic search identified a total of 70 metabolites related to oxidative stress, each showing significant differences in CNS disease patients compared with healthy controls. The identified metabolites were classified into six major classes: lipids, amino acids, nucleosides, organic acids, sugars, alcohols, and other molecules. As shown in Fig. 1, lipids were the most reported group, with 23 entities identified. Although all metabolites were quantified using GC-MS, analytical methodologies are not fully standardized, and sample handling, preparations techniques, extraction procedures and derivatization methods varied among studies, which need to be taken into consideration when comparing results.

#### 3.2.1 Lipids

Altered lipid metabolites are summarized in Table 3. Markers of lipid peroxidation, including 4-hydroxy-2-nonenal, 4-oxo-2-nonenal, malondialdehyde, isoprostanes, isofurans, and neuroprostanes, were consistently elevated across multiple CNS diseases, compared with healthy controls, in both blood and cerebrospinal fluid. Significantly elevated levels of these established oxidative stress markers were observed in tick-borne encephalitis, with increased levels of 4-hydroxy-2-nonenal, malondialdehyde, and neuroprostanes [35,36]; and in delayed cerebral ischemia, with increased levels of isoprostanes and isofurans [37]. Additionally, neuroprostanes, generated from the oxidation of docosahexaenoic acid, were found to be increased in traumatic brain injury [38], multiple sclerosis, autism, Rett syndrome, and Down syndrome [39].

**Table 3:** Lipids and related molecules linked to oxidative stress found significantly dysregulated in CNS diseases.

Lipid		Disease	Sample	Reference
**Lipid peroxidation products and biomarkers**				
4-hydroxy-2-nonenal	>	Tick-borne encephalitis (TBE)	Blood plasma	Dobrzyńska 2022
	>	TBE	Cerebrospinal fluid (CSF)	Groth 2023
4-oxo-2-nonenal	>	Subarachnoid hemorrhage	Blood	Jarocka-Karpowicz 2020
Malondialdehyde	>	TBE	Blood plasma	Dobrzyńska 2022
	>	TBE	CSF	Groth 2023
Isoprostanes	>	Traumatic brain injury (TBI)	Blood plasma	Yen 2015
	>	Delayed cerebral ischemia	CSF	Gomes 2022
Isofurans	>	Delayed cerebral ischemia	CSF	Gomes 2022
Neuroprostanes	>	TBE	Blood plasma	Dobrzyńska 2024
	>	TBI	Blood plasma	Yen 2015
	>	Multiple sclerosis (MS)	Blood plasma	Signorini 2018
	>	Autism		
	>	Rett syndrome		
	>	Down syndrome		
**Oxysterols**				
7β-hydroxycholesterol	>	Alzheimer's disease (AD)	Blood plasma	Zarrou 2020
	>	Huntington's disease (HD)	Postmortem brain tissue	Kreilaus 2016
24S-hydroxycholesterol	>	AD	Blood plasma	Zarrou 2020
	<	HD	Postmortem brain tissue	Kreilaus 2016
25-hydroxycholesterol	>	AD	Blood plasma	Zarrou 2020
7-oxo-cholesterol	>	HD	Postmortem brain tissue	Kreilaus 2016
Cholestane-3β,5α,6β-triol (C-triol)	>	Niemann-Pick type C disease	Blood plasma	Kannenberg 2017
**Dicarboxylic acids, products of fatty acid oxidation**				
Azelaic acid	>	AD, presymptomatic	Urine	Castor 2020
Pimelic acid	>	AD, presymptomatic	Urine	Castor 2020
Suberic acid	>	AD, presymptomatic	Urine	Castor 2020
**Phospholipids and acylcarnitines**				
Phosphatidylcholine (PC)	>	MS, drug-naïve	Blood serum	Andersen 2019
PC ae 40:5	>	MS, drug-naïve	Blood serum	Andersen 2019
PC ae 42:5	>	MS, drug-naïve	Blood serum	Andersen 2019
C14:1 acylcarnitine	>	MS, drug-naïve	Blood serum	Andersen 2019
**Other lipids**				
Oleic acid	>	AD	Blood serum	González-Domínguez 2015
Elaidic acid	>	Chronic fatigue in adolescents	Urine	Zhao 2022
Palmitic acid	>	PD	Blood plasma	Glaab 2019
	<	AD	Blood serum	González-Domínguez 2015
**Oxidation-sensitive PUFAs**				
Linoleic acid	<	Methamphetamine dependence	Peripheral blood mononuclear cells	Su 2022

Significant differences in the levels of 7β-hydroxycholesterol, 24S-hydroxycholesterol, 25-hydroxycholesterol and 7-oxocholesterol were observed in the blood of patients with Alzheimer's disease [40] and in the cerebral tissue of patients with Huntington's disease [41]. These alterations suggest increased inflammatory signaling, apoptosis, and oxidative damage in the CNS and vascular system. While all of these oxysterols were increased, 24S-hydroxycholesterol levels were decreased in Huntington's disease [41].

Azelaic acid, pimelic acid, and suberic acid, all dicarboxylic acids derived from lipid peroxidation, were found to be significantly increased in the urine of patients with Alzheimer's disease [42]. These elevations may indicate augmented oxidative stress, mitochondrial dysfunction, and impaired fatty acid metabolism.

The phospholipids phosphatidylcholine (PC), PC ae C40:5, PC ae C42:5 and PC ae C14:1 acylcarnitine were all found to be elevated in the blood serum of patients with multiple sclerosis [43].

#### 3.2.2 Amino acids and derivatives

We found significant changes in the levels of specific amino acids across different human samples and CNS diseases, as shown in Table 4. Some amino acids with protective roles against oxidative stress were altered, such as glutamine in multiple sclerosis [44], methamphetamine dependence [45], and Alzheimer's disease [46]. Glycine was also found to be dysregulated in acute ischemic stroke [47], while cysteine was disrupted in autism [48] and methamphetamine dependence [45], as well as cystine in Alzheimer's disease [46].

**Table 4:** Amino acids and related compounds linked to oxidative stress, significantly dysregulated in CNS diseases.

Amino acid		Disease	Sample	Reference
**Protective to oxidative stress**				
Glutamine	>	Multiple sclerosis (MS)	Blood plasma	Poddighe 2017
	>	Methamphetamine dependence	Peripheral blood mononuclear cells (PBMCs)	Su 2022
	<	Alzheimer's disease (AD)	Blood serum	González-Domínguez 2015
Cystine	<	AD	Blood serum	González-Domínguez 2015
Cysteine	<	Methamphetamine dependence	PBMCs	Su 2022
	>	Autism	Urine	Mussap 2020
Glycine	<	Acute ischemic stroke	Blood serum	Wang 2017
Serine	>	Fibromyalgia	Blood plasma	Piras 2022
	<	Acute ischemic stroke	Blood serum	Wang 2017
	<	Obstructive sleep apnea	Sweat	Castillo-Peinado 2024
Aspartic acid	>	Methamphetamine dependence	PBMCs	Su 2022
	<	AD	Blood serum	González-Domínguez 2015
	<	Fibromyalgia	Blood plasma	Piras 2022
Histidine	<	AD	Blood serum	González-Domínguez 2015
Tryptophan	>	Autism	Urine	Mussap 2020
	>	Fatigue in patients with rheumatoid arthritis	Blood plasma	Surowiec 2016
	<	Acute ischemic stroke	Blood serum	Wang 2017
	<	AD	Blood serum	González-Domínguez 2015
**Contributor or indicator of oxidative stress**				
Pyroglutamate	>	MS, drug-naïve	Blood serum	Andersen 2019
	>	Obstructive sleep apnea	Sweat	Castillo-Peinado 2024
	<	AD	Blood serum	González-Domínguez 2015
Glutamate	>	MS	Blood plasma	Poddighe 2017
	<	Acute ischemic stroke	Blood serum	Wang 2017
Homocysteine	>	Methamphetamine dependence	PBMCs	Su 2022
Aminomalonic acid	<	Autism	Urine	Mussap 2020
	>	Posttraumatic stress disorder	Blood plasma	Konjevod 2021

Amino acids and related compounds that can act as indicators or contributors to oxidative stress, including pyroglutamate, glutamate, homocysteine, and aminomalonic acid, were also found to be dysregulated. For example, pyroglutamate was elevated in blood in drug-naïve patients with multiple sclerosis [43] but reduced in patients with Alzheimer's disease [46].

#### 3.2.3 Nucleosides, nucleotides, and related bases

Several purine metabolites, presented in Table 5, exhibited disease-dependent shifts reflecting impaired nucleotide turnover and oxidative stress responses. Uric acid, an endogenous antioxidant, was increased in the urine of adolescents with chronic fatigue symptoms [49] yet decreased in the blood of patients with Alzheimer's disease [46] and autism [48].

**Table 5:** Nucleotides, nucleosides, and related molecules related to oxidative stress and altered in CNS diseases.

Nucleotides and related molecules		Disease	Sample	Reference
**Purine derivatives & nucleosides**				
Uric acid	<	Alzheimer's disease (AD)	Blood serum	González-Domínguez 2015
	<	Autism	Urine	Mussap 2020
	>	Chronic Fatigue Symptoms in Adolescents	Urine	Zhao 2022
Hypoxanthine	>	Methamphetamine dependence	Peripheral blood mononuclear cells (PBMCs)	Su 2022
	<	Acute Ischemic Stroke	Blood serum	Wang 2017
Xanthine	>	Methamphetamine dependence	PBMCs	Su 2022
Inosine	<	Chronic Fatigue Symptoms in Adolescents	Urine	Zhao 2022
Guanosine	<	Methamphetamine dependence	PBMCs	Su 2022
Adenosine	>	AD	Blood serum	González-Domínguez 2015
Purine	<	Acute ischemic stroke	Blood serum	Wang 2017
**Oxidative DNA damage products**				
2,6-diamino-4-hydroxy-5-formamidopyrimidine	>	Bipolar disorder	Blood leukocytes	Ceylan 2018
5-hydroxy-5-methylhydantoin	>	Bipolar disorder	Blood leukocytes	Ceylan 2018
Thymine glycol	>	Bipolar disorder	Blood leukocytes	Ceylan 2018

Inosine and guanosine, both considered neuroprotective [50], were found to be decreased in two conditions: inosine in the urine of adolescents with chronic fatigue [49], and guanosine in peripheral blood mononuclear cells in patients with methamphetamine dependence [45]. Conversely, adenosine, an anti-inflammatory purine, was increased in the blood of patients with Alzheimer's disease [46].

Hypoxanthine and xanthine, intermediates that can generate ROS via xanthine oxidase [51], were elevated in patients with methamphetamine dependence [45] but reduced in acute ischemic stroke [47]. Total purine levels were also reduced in acute ischemic stroke, consistent with cellular energy depletion [47].

Finally, three markers of DNA base oxidation, 2,6-diamino-4-hydroxy-5-formamidopyrimidine, 5-hydroxy-5-methylhydantoin, and thymine glycol, were consistently elevated in blood leukocytes of patients with bipolar disorder [52], indicating systemic oxidative DNA damage and potential genomic instability in mood disorders.

#### 3.2.4 Organic acids

Metabolites related to mitochondrial and glycolytic pathways, as well as glutathione cycling, summarized in Table 6, showed disease-specific but interconnected alterations across CNS conditions.

**Table 6:** Organic acids related to oxidative stress and altered in CNS diseases.

Organic acid		Disease	Sample	Reference
**TCA cycle intermediates**				
Succinic acid	<	Alzheimer's disease (AD), presymptomatic	Urine	Castor 2020
Glutaric acid	<	AD, presymptomatic	Urine	Castor 2020
α-ketoglutarate	>	AD	Blood serum	González-Domínguez 2015
	<	Methamphetamine dependence	Peripheral blood mononuclear cells (PBMCs)	Su 2022
Fumaric acid	<	Methamphetamine dependence	PBMCs	Su 2022
Isocitric acid	>	AD	Blood serum	González-Domínguez 2015
Malic acid	>	Posttraumatic stress disorder	Blood plasma	Konjevod 2021
Citrate	<	Acute ischemic stroke	Blood serum	Wang 2017
**Glycolytic end products**				
Pyruvate	>	Obstructive sleep apnea	Sweat	Castillo-Peinado 2024
	<	Fibromyalgia with self-reported electromagnetic sensitivity	Blood plasma	Piras 2022
	<	Methamphetamine dependence	PBMCs	Su 2022
Lactic acid	>	AD	Blood serum	González-Domínguez 2015
	>	Acute ischemic stroke	Blood serum	Wang 2017
**Glutathione metabolism**				
Pyroglutamic acid (5-oxoproline)	>	Multiple sclerosis (MS), drug-naïve	Blood serum	Andersen 2019
	<	AD (newly diagnosed of sporadic AD, that had not yet received any type of medication)	Blood serum	González-Domínguez 2015
	<	MS	Blood plasma	Poddighe 2017

Key intermediates of the tricarboxylic acid (TCA) cycle, such as succinic acid, citrate, and fumaric acid, were decreased in presymptomatic Alzheimer's disease [42], acute ischemic stroke [47], and methamphetamine dependence [45], pointing to impaired mitochondrial flux and energy depletion. In contrast, α-ketoglutarate, isocitric acid, and malic acid were increased in Alzheimer's disease [46] and posttraumatic stress disorder [53], suggesting compensatory anaplerotic activity.

These shifts suggest that TCA intermediates tend to accumulate in neurodegeneration (possibly reflecting anaplerotic flux) but are depleted in substance dependence and acute ischemia, consistent with impaired mitochondrial energy production.

Glycolytic end products displayed similar context-dependent trends. Lactic acid was elevated in Alzheimer's disease [46] and acute ischemic stroke [47], consistent with increased anaerobic glycolysis. Pyruvate levels, in turn, were increased in the sweat of patients with obstructive sleep apnea [54] but decreased in fibromyalgia [55] and methamphetamine dependence [45].

Pyroglutamic acid, an indicator of glutathione cycle activity, showed disease- and treatment-dependent variation: it was increased in drug-naïve multiple sclerosis [43] but decreased in Alzheimer's disease [46] and in treated multiple sclerosis patients [44], reflecting context-specific alterations in glutathione turnover under oxidative stress.

Collectively, these metabolite shifts highlight a coherent picture of mitochondrial dysfunction, altered glycolytic flux, and impaired antioxidant cycling across neurodegenerative, inflammatory, and stress-related CNS disorders.

#### 3.2.5 Sugars and alcohols

Multiple sugars, sugar alcohols, and related metabolites showed significant alterations in CNS conditions, as depicted in Table 7, pointing to disturbances in carbohydrate metabolism, polyol pathway activity, and redox imbalance.

**Table 7:** Sugars, alcohols, and related metabolites related to oxidative stress, altered in CNS diseases.

Sugars and alcohols		Disease	Sample	Reference
Glucose	>	Alzheimer's disease	Blood serum	González-Domínguez 2015
Fructose	>	Posttraumatic stress disorder	Blood plasma	Konjevod 2021
	>	Parkinson's disease	Cerebrospinal fluid (CSF)	Trezzi 2017
	<	Multiple sclerosis (MS)	Blood plasma	Poddighe 2017
Myo-inositol	>	MS, drug-naïve	Blood serum	Andersen 2019
	<	MS	Blood plasma	Poddighe 2017
	<	Methamphetamine dependence	Peripheral blood mononuclear cells (PBMCs)	Su 2022
	<	Autism	Urine	Mussap 2020
Sorbitol	<	Methamphetamine dependence	PBMCs	Su 2022
Threonic acid	>	Parkinson's disease	CSF	Trezzi 2017
	<	MS	Blood plasma	Poddighe 2017
6-phosphogluconic acid	>	Chronic Fatigue Symptoms in Adolescents	Urine	Zhao 2022
Erythronic acid	<	Acute Ischemic Stroke	Blood serum	Wang 2017
Dehydroascorbic acid	<	Parkinson's disease	CSF	Trezzi 2017

Blood glucose was significantly increased in patients with Alzheimer's disease [46], likely reflecting impaired cerebral glucose uptake or insulin signaling, both key mechanisms implicated in Alzheimer's disease pathogenesis. Fructose levels were significantly increased in the blood of patients with posttraumatic stress disorder [53] and in cerebrospinal fluid of patients with Parkinson's disease [56], suggesting upregulated polyol pathway flux or altered fructose metabolism. Conversely, blood fructose was decreased in multiple sclerosis [44]. These contrasting trends suggest disease-specific differences in polyol pathway activity and fructose clearance.

Myo-inositol, a key osmolyte and marker of glial activity [57], was elevated in the blood of patients with multiple sclerosis [43], consistent with glial activation. In contrast, it was decreased in urine from patients with autism [48], in methamphetamine dependence [45], and in the blood of another multiple sclerosis cohort [44], suggesting context-specific dysregulation.

Additional sugar-related metabolites, including sorbitol, threonic acid, erythronic acid, dehydroascorbic acid, and 6-phosphogluconic acid, were also found to be dysregulated in some CNS conditions [44,45,47,49,56]. These alterations reveal further disruptions in polyol metabolism, oxidative vitamin C turnover, and pentose phosphate pathway activity.

#### 3.2.6 Other molecules

Other metabolites associated with oxidative stress and metabolic dysregulation, presented in Table 8, showed significant alterations across different CNS conditions. Phosphate, which is an essential mineral [58], was reduced in patients with multiple sclerosis [44]. Nitrite and nitrate, reflecting nitric oxide turnover, were elevated in attention-deficit/hyperactivity disorder [59]. The dopamine metabolite 3,4-dihydroxybenzene acetic acid and epinephrine were increased in the urine of chronic fatigue patients [49], consistent with augmented dopamine catabolism and sympathoadrenal activation. Urea levels showed mixed trends in blood across conditions, with alterations reported in Parkinson's disease [60], fibromyalgia with self-reported electromagnetic sensitivity [55], Alzheimer's disease [46], and acute ischemic stroke [47].

**Table 8:** Other compounds related to oxidative stress and significantly altered in CNS diseases.

Other compounds		Disease	Sample	Reference
Phosphate	<	Multiple sclerosis (MS)	Blood plasma	Poddighe 2017
3,4-dihydroxybenzeneacetic acid	>	Chronic fatigue in adolescents	Urine	Zhao 2022
Epinephrine	>	Chronic fatigue in adolescents	Urine	Zhao 2022
Nitrate NO_3_^−^	>	Attention-deficit/hyperactivity disorder (ADHD)	Blood plasma	Sinningen 2023
Nitrite NO_2_^−^	>	ADHD	Blood plasma	Sinningen 2023
Quinic acid	>	Autism	Urine	Mussap 2020
Urea	>	Parkinson's disease	Blood plasma	Glaab 2019
	>	Fibromyalgia with self-reported electromagnetic sensitivity	Blood plasma	Piras 2022
	<	Alzheimer's disease	Blood serum	González-Domínguez 2015
	<	Acute ischemic stroke	Blood serum	Wang 2017
Indoleacetic acid	<	Fatigue in patients with rheumatoid arthritis	Blood plasma	Surowiec 2016
	>	Autism	Urine	Mussap 2020

## 4 Discussion

This systematic review synthesizes evidence from 24 human studies using GC-MS to investigate metabolomic alterations associated with oxidative stress across CNS disorders. A total of 70 metabolites were identified as significantly altered in patients compared with healthy controls, spanning a wide range of biochemical classes, including lipids, amino acids, nucleotides, organic acids, sugars, and other small molecules.

Neurodegenerative conditions such as Alzheimer's disease, Parkinson's disease, multiple sclerosis, and Huntington's disease were the most frequently investigated, accounting for 10 out of 24 studies. Blood was the most commonly analyzed sample, with 14 studies, reflecting its accessibility and systemic relevance.

The identified metabolites reflect disturbances in core cellular processes, including redox balance, lipid peroxidation, nucleotide turnover, and mitochondrial energy metabolism. These findings provide a comprehensive overview of oxidative stress-linked metabolic dysregulation in CNS disorders and support the existence of shared pathophysiological mechanisms that may transcend traditional diagnostic categories.

### 4.1 GC-MS

GC-MS provides high sensitivity, specificity, and reproducibility for detecting small, volatile, and thermally stable compounds [24], including organic acids, amino acids, sugars, and lipid peroxidation products, many of which are key indicators of oxidative damage. Furthermore, GC-MS has the advantage of being a mature, standardized platform with extensive metabolite libraries, facilitating compound identification and cross-study comparisons [23]. The studies included in the review consistently demonstrated that GC-MS can provide robust, high-resolution metabolic profiling from diverse human biofluids, facilitating the detection of altered metabolites with potential diagnostic and mechanistic relevance.

### 4.2 Oxidative stress and CNS diseases

Oxidative stress is widely recognized as a central pathological mechanism in neurodegenerative and other CNS disorders [15]. The brain is particularly susceptible to oxidative damage due to its high metabolic rate, abundant polyunsaturated lipid content, and relatively low levels of endogenous antioxidant defenses [14], making neuronal and glial cells highly sensitive to ROS, as seen in diseases such as Alzheimer's [14], Parkinson's, or Huntington's [15,61]. Excessive ROS can contribute to synaptic dysfunction, protein aggregation, mitochondrial failure, and ultimately neuronal death through pathways such as apoptosis, ferroptosis, and autophagy [61]. Notably, oxidative stress not only contributes to neuronal injury but also promotes neuroinflammation via activation of microglia and astrocytes and dysregulation of immune signaling, which can create a self-perpetuating cycle of damage [62]. This widespread oxidative burden is detectable in CNS tissue as well as in peripheral biofluids, where metabolites reflecting oxidative damage and impaired redox cycling, such as lipid peroxidation products, glutathione precursors, and oxidized DNA bases, are consistently altered across CNS conditions.

### 4.3 Lipid oxidation and membrane damage

Lipid oxidation products were elevated not only in CNS infection and brain injury, where classical lipid peroxidation products were increased [35,37,38,63,64], but also in neurodegenerative diseases, with increased oxysterols [40,41] and dicarboxylic acids [42], and in autism, with increased neuroprostanes [39]. These markers were consistently elevated across different sample types, including blood, cerebrospinal fluid, urine, and brain tissue. Neuroprostanes, which are non-enzymatic products of oxidized docosahexaenoic acid, a major polyunsaturated fatty acid in neuronal membranes, have previously been found to be increased in brain tissue and cerebrospinal fluid in neurodegenerative diseases and interpreted as markers of neuronal damage [65]. Different works have shown that the increase of these molecules under CNS insult also reflects in blood, as it has been observed here in tick-borne encephalitis [36], traumatic brain injury [38], multiple sclerosis, and Rett syndrome, but also in Down syndrome and autism [39], consistently showing higher levels in specific neuroprostanes compared with healthy controls.

Additional evidence of membrane damage is provided by the consistent increase in urinary dicarboxylic acids, including azelaic, suberic, and pimelic acids. These compounds likely arise from increased ω-oxidation, which could be a compensatory pathway for fatty acid metabolism in the context of impaired β-oxidation [66,67] due to oxidative stress or energy impairment. In presymptomatic Alzheimer's, increases in urinary dicarboxylic acids correlated with lower hippocampal volume and elevated cerebrospinal fluid tau protein [42] highlighting their potential link to early neurodegeneration. Urine dicarboxylic acid profiles have been proposed as candidate biomarker for Alzheimer's disease in other works [68]. Similar urinary increases have also been reported in other pathologies, including diabetes [69] and autism [70], indicating a broader systemic involvement of oxidative lipid metabolism in disease.

The oxidation-sensitive polyunsaturated fatty acid linoleic acid was decreased in methamphetamine dependence [45]. In contrast, oleic acid, a monounsaturated fatty acid, was increased in Alzheimer's disease [46], though its precise role in CNS pathology remains to be clarified.

Phospholipid remodeling was also evident, with elevated levels of PC species (PC ae C40:5, PC ae C42:5) reported in multiple sclerosis patients [43]. Given that phosphatidylcholines are fundamental components of cellular membranes and myelin sheaths, their dysregulation may indicate underlying membrane instability. Notably, the increased levels of these phosphatidylcholines were associated with upregulated expression of cytosolic phospholipase A2 group IV C (PLA2G4C), an enzyme that hydrolyzes glycerophospholipids to release free fatty acids and lysophospholipids, both of which serve as precursors in the production of signaling molecules [43]. This upregulation may represent a pathological attempt at membrane repair or remodeling in response to oxidative stress.

Among saturated fatty acids, palmitic acid showed disease-specific alterations: it was increased in Parkinson's disease [60], potentially contributing to lipotoxicity and inflammation, but decreased in Alzheimer's disease [46], suggesting divergent lipid metabolic adaptations. The trans fatty acid elaidic acid, with well-documented pro-oxidant and pro-inflammatory effects [[71], [72], [73]], was increased in adolescents with chronic fatigue symptoms [49] possibly reflecting diet-related metabolic stress or impaired lipid clearance.

Collectively, these findings highlight a convergent pattern of oxidative lipid damage, altered fatty acid metabolism, and altered membrane remodeling across CNS disorders, which may underlie neuronal vulnerability and progressive functional decline.

### 4.4 Redox imbalance and antioxidant depletion

Several metabolites involved in glutathione metabolism, including glutamine, cystine, cysteine, and glycine, were found to be dysregulated across CNS conditions such as Alzheimer's disease [46], multiple sclerosis [44], autism [48], and methamphetamine dependence [45]. These alterations suggest compromised glutathione cycling and a reduced buffering capacity against ROS.

Pyroglutamic acid, a by-product of the γ-glutamyl cycle, was increased in the blood of drug-naïve multiple sclerosis [43] and in the sweat of obstructive sleep apnea [54], but decreased in the blood of Alzheimer's disease patients [46]. Similarly, glutamate, a key metabolite in excitatory neurotransmission [74], was elevated in multiple sclerosis [44] whereas reduced in acute ischemic stroke [47], pointing to disrupted glutamate homeostasis and potential excitotoxicity. Aberrant glutamate homeostasis, which can be reflected by altered levels in glutamine, glutamate and pyroglutamate, can contribute to excitotoxicity and axonal injury, a well-known phenomenon in CNS disorders such as multiple sclerosis [75].

Additionally, homocysteine, a sulfur-containing amino acid widely recognized as a biomarker for various conditions, including cardiovascular diseases and neurological disorders [76], was found to be elevated in peripheral blood mononuclear cells from individuals with methamphetamine dependence [45]. Homocysteine accumulation is known to impair methylation processes, promote oxidative stress, and exacerbate neurotoxicity [76], further linking redox imbalance to disease pathology.

### 4.5 Disturbed nucleotide turnover and DNA oxidation

Alterations in purine metabolism and oxidative DNA damage markers observed across several CNS diseases in this review underscore the intricate link between nucleotide turnover, oxidative stress, and neurodegeneration. Uric acid, a terminal product of purine catabolism and a potent endogenous antioxidant [77], was decreased in the blood of patients with Alzheimer's disease [46] and in the urine of autistic children [48], but elevated in the urine of adolescents with chronic fatigue symptoms [49], possibly reflecting a compensatory response to heightened oxidative stress. This pattern suggests that uric acid may act as a dynamic buffer against oxidative damage, with its levels varying according to disease stage or compensatory responses to redox imbalance.

Neuroprotective purine nucleosides, inosine [78] and guanosine [79], were decreased in the urine of adolescents with chronic fatigue [49] and in peripheral blood mononuclear cells from methamphetamine-dependent individuals [45], respectively. These nucleosides possess anti-inflammatory and neurotrophic properties, and their depletion may reflect increased purine degradation, reduced salvage pathway activity, or the exhaustion of protective purine reserves under chronic oxidative or metabolic stress. Adenosine, another purine with anti-inflammatory and neuromodulatory functions [80], was increased in the blood of patients with Alzheimer's disease [46], potentially reflecting a compensatory response to neuronal injury.

Hypoxanthine and xanthine, intermediates in purine degradation that generate ROS through xanthine oxidase activity [81], were elevated in methamphetamine dependence [45], consistent with chronic upregulation of ROS-generating pathways, yet reduced in acute ischemic stroke [47], highlighting a contrast between chronic oxidative upregulation and acute purine depletion due to energy failure.

Additionally, markers of oxidative DNA damage, including 2,6-diamino-4-hydroxy-5-formamidopyrimidine [82], 5-hydroxy-5-methylhydantoin [83], and thymine glycol [84], were consistently elevated in the leukocytes of patients with bipolar disorder [52]. These compounds are well-established markers of oxidative DNA damage, often resulting from hydroxyl radical attack, and their accumulation suggests persistent oxidative stress and potentially impaired DNA repair mechanisms in mood disorders.

Altogether, these findings indicate a disruption in nucleotide balance, involving both catabolic and salvage pathways, alongside direct oxidative damage to nucleic acids. This convergence may contribute to genomic instability, inflammation, and progressive neurodegeneration.

### 4.6 Mitochondrial dysfunction and energy metabolism

Multiple metabolites identified across CNS-related conditions converge on disrupted mitochondrial function and energy metabolism as central features of disease pathophysiology. This systematic review identified articles showing significant reductions in key TCA cycle intermediates, including glutaric and succinic acid in presymptomatic Alzheimer's disease [42], fumaric acid in methamphetamine dependence [45], and citric acid in acute ischemic stroke [47]. These reductions likely reflect compromised mitochondrial oxidative phosphorylation and diminished metabolic flux through the TCA cycle under acute or toxic stress. In contrast, elevations in α-ketoglutarate, isocitric acid, and malic acid in Alzheimer's disease [46] and posttraumatic stress disorder [53] may indicate compensatory anaplerotic responses or a shift toward altered mitochondrial substrate utilization.

Shifts in glycolytic end products also support the presence of energy pathway reprogramming. Lactic acid was elevated in blood of patients with Alzheimer's disease and acute ischemic stroke [46,47], consistent with enhanced anaerobic glycolysis secondary to mitochondrial impairment. Disease-specific pyruvate changes, elevated in obstructive sleep apnea [54] but reduced in fibromyalgia [55] and methamphetamine dependence [45], indicate altered glycolytic flux and systemic metabolic adaptation.

Mechanistically, oxidative stress and mitochondrial dysfunction form a vicious cycle. Mitochondria are both a major source and target of ROS; excessive ROS impairs mitochondrial DNA, lipids, and respiratory chain complexes, particularly complexes I and III [11]. This leads to ATP depletion, electron leakage, and further ROS production. Oxidative impairment of the TCA cycle, β-oxidation, and oxidative phosphorylation promotes the accumulation of metabolic intermediates and triggers alternative pathways such as ω-oxidation, as evidenced by urinary dicarboxylic acid elevations [42].

These findings underscore mitochondrial dysfunction as both a hallmark and a driver of CNS pathology, with oxidative stress acting as a key amplifier of bioenergetic failure, neuroinflammation, and neuronal degeneration.

#### 4.6.1 Sugar and polyol pathway dysregulation

Dysregulation of sugar and sugar alcohol metabolism emerged as a common feature across several CNS-related conditions, pointing to disruptions in carbohydrate utilization, insulin signaling, glial function, and oxidative stress-related pathways. Elevated blood glucose levels in patients with Alzheimer's disease [46] may reflect impaired cerebral glucose uptake or peripheral insulin resistance, both of which are well-documented contributors to Alzheimer's disease pathogenesis and oxidative stress [14,85]. Altered glucose metabolism limits neuronal energy supply and forces greater reliance on anaerobic pathways, thereby increasing ROS production, mitochondrial dysfunction, and neuronal vulnerability [85].

Fructose, a key polyol pathway intermediate, which, when consumed in excess, can promote systemic oxidative stress and inflammation [[86], [87], [88]], showed disease-specific trends, being elevated in the blood of patients with posttraumatic stress disorder [53] and in the cerebrospinal fluid of patients with Parkinson's disease [56], but decreased in the blood of patients with multiple sclerosis [44]. These findings suggest differential regulation of aldose reductase and sorbitol dehydrogenase, enzymes that convert glucose to sorbitol and then to fructose. Overactivation of this pathway under oxidative stress consumes NADPH, thereby limiting glutathione regeneration and thereby weakening antioxidant defense [89].

Myo-inositol, involved in glial signaling and phosphoinositide metabolism [90], was altered in multiple sclerosis [43,44], methamphetamine dependence [45], and autism [48], potentially reflecting glial activation or astrocytic dysfunction [91].

Other sugar-related metabolites, such as sorbitol [45], erythronic acid [47], threonic acid, dehydroascorbic acid [56], and 6-phosphogluconic acid [49], point to broader alterations in the polyol pathway, oxidative vitamin C metabolism, and the pentose phosphate pathway. Since the pentose phosphate pathway is critical for NADPH production, its dysregulation may further compromise antioxidant capacity [92].

Together, these findings point to disturbances in sugar and sugar alcohol metabolism as central contributors to CNS pathophysiology through redox imbalance, mitochondrial stress, and neuroinflammation. Their disease-specific patterns also highlight potential applications in biomarker discovery and metabolic targeting for therapeutic intervention.

### 4.7 Other oxidative stress metabolites

Beyond core disruptions in lipid, energy, and nucleotide metabolism, other small molecules associated with oxidative stress showed disease-specific alterations across CNS conditions.

Reduced phosphate, an essential mineral for pH buffering, signal transduction, energy metabolism, and component of several molecules and biological structures [58], was decreased in the blood of patients with multiple sclerosis [44], which may reflect impaired ATP turnover or mitochondrial dysfunction. Nitrite and nitrate, end-products of nitric oxide metabolism [93], were elevated in attention-deficit/hyperactivity disorder [59], suggesting dysregulated nitric oxide signaling. While nitric oxide plays physiological roles in vasodilation and synaptic plasticity, excessive production or uncoupled nitric oxide synthase activity can lead to nitrosative stress via peroxynitrite formation, contributing to neuroinflammation and oxidative damage [94].

Alterations in neurotransmitter-related metabolites, such as elevated levels of 3,4-dihydroxyphenylacetic acid (a dopamine catabolite) and epinephrine in patients with chronic fatigue symptoms [49], point to augmented dopamine turnover and sympathoadrenal activation, consistent with chronic stress. These changes may not only reflect neurochemical imbalances but also intersect with redox balance, as catecholamine oxidation generates ROS and toxic quinone species [95].

Urea, a marker of nitrogen metabolism, showed heterogeneous trends: elevated in Parkinson's disease [60] and in fibromyalgia with self-reported electromagnetic sensitivity [55], but decreased in Alzheimer's disease [46] and acute ischemic stroke [47]. These variations may reflect disease-specific changes in protein metabolism, potentially driven by mitochondrial or renal involvement, differential muscle catabolism, or systemic inflammation and metabolic status [96].

Taken together, these findings reinforce the idea that oxidative stress in CNS diseases is not limited to traditional markers of lipid or DNA oxidation. Instead, it involves broader disturbances in redox signaling, nitrogen metabolism, catecholamine turnover, and mitochondria-linked pathways.

### 4.8 Clinical implications and biomarker potential

The metabolomic alterations identified in this review have significant clinical relevance, particularly for the development of diagnostic biomarkers and therapeutic strategies targeting oxidative stress in CNS diseases. GC-MS–based metabolomics offers high sensitivity and specificity for the detection of oxidative stress–related metabolites in accessible biofluids such as blood and urine, offering a powerful tool to non-invasively capture systemic redox imbalances.

The consistent changes in metabolites associated with lipid peroxidation (e.g., neuroprostanes, isoprostanes, oxysterols), antioxidant depletion (e.g., cystine, pyroglutamate, dehydroascorbic acid), and mitochondrial dysfunction (e.g., TCA intermediates, acylcarnitines) across diverse CNS conditions support the notion that oxidative stress is a core and converging mechanism. The presence of these alterations in easily accessible biofluids such as blood and urine further strengthens their potential as non-invasive biomarkers for both disease detection and monitoring.

Some of these metabolites also exhibit disease-specific trends, offering potential for patient stratification. For instance, 24S-hydroxycholesterol reflects neuronal cholesterol turnover and may serve as an early marker of Alzheimer's pathology, whereas increased dicarboxylic acids in urine may indicate systemic mitochondrial stress. Similarly, reductions in uric acid and glutathione precursors could help identify patients with impaired antioxidant defense, potentially guiding redox-targeted interventions.

Nevertheless, methodological variability, encompassing differences in sample handling, extraction, derivatization, and chromatographic conditions, can significantly impact the quantification of labile or redox-sensitive metabolites, such as glutathione, cysteine, or lipid peroxidation products, which are susceptible to *ex vivo* oxidation and artefactual conversion. To enhance reproducibility and facilitate cross-study comparability, future research should prioritize the standardization of analytical workflows and the transparent reporting of pre-analytical variables. The harmonization of GC–MS protocols, enabling data normalization strategies, will be crucial for establishing reproducible redox-metabolite signatures and advancing clinical translation.

These findings reinforce precision medicine strategies aimed at restoring redox homeostasis, such as antioxidant therapies, modulators of mitochondrial metabolism, or dietary interventions. Integration of metabolite panels into clinical workflows, together with longitudinal validation, could enhance early detection, improve disease monitoring, and enable personalized interventions across a spectrum of CNS disorders.

## 5 Limitations

This systematic review presents limitations that should be acknowledged when interpreting the findings. First, there was considerable heterogeneity in study designs, including patient populations, disease stages, sample types, and control groups, which may have influenced the reported metabolite alterations. These differences complicate cross-study comparisons, as metabolite levels vary across biofluids and are influenced by age, comorbidities, and clinical status.

A key limitation is the lack of longitudinal data and standardization across GC-MS methodologies. Variations in GC-MS protocols, including derivatization, sample handling, extraction procedures, and the use of targeted versus untargeted approaches limit metabolite comparability. Consequently, even among studies of the same disease, there was limited overlap in reported metabolites, reducing the ability to identify robust, disease-specific signatures. The predominance of targeted panels further highlights the need for more untargeted and standardized metabolomic profiling.

The relatively small number of studies for some diseases and the absence of longitudinal data restrict conclusions about temporal changes and disease progression. Moreover, most studies did not adjust for potential confounding factors such as medication use, diet, or physical activity—all of which can significantly impact metabolic profiles.

Additional limitations include the lack of or differences in normalization strategies across studies, such as adjusting urinary metabolites to creatinine or total protein, which can improve interpretability, as illustrated in links between dicarboxylic acids and hippocampal atrophy (Castor 2020). Finally, differences between metabolite levels in tissues, cells, and circulating fluids may obscure localized redox alterations, underscoring the need for integrative, multi-compartment studies in future research.

## 6 Conclusions

This systematic review highlights the utility of GC-MS in identifying metabolomic alterations associated with oxidative stress across a broad spectrum of CNS diseases. A total of 70 metabolites were found to be significantly altered in patients compared with healthy controls, reflecting disruptions in lipid metabolism, energy pathways, nucleotide turnover, redox balance, and mitochondrial function.

The findings underscore oxidative stress as a converging pathological mechanism across diverse CNS disorders. Several metabolites, including lipid peroxidation products, TCA cycle intermediates, and glutathione-related amino acids, emerged as consistently dysregulated and may serve as non-invasive biomarkers for diagnosis, disease monitoring, or therapeutic targeting.

Despite heterogeneity across studies and methodological variability, the consistent detection of oxidative stress-linked metabolites supports the integration of GC-MS–based metabolomics into future clinical and research frameworks. Standardization of protocols and longitudinal, untargeted approaches will be critical for validating these signatures and translating them into precision medicine strategies.

In summary, GC-MS metabolomics offers valuable insights into systemic and CNS-specific redox alterations, thereby opening new avenues for understanding disease mechanisms and developing targeted interventions in CNS pathology.

## Funding sources

Support for this work was provided by the National Institute of 10.13039/100018696Health/Instituto de Salud Carlos III-10.13039/501100004587ISCIII, Spain (PI22/00443 and DTS24/00141 to 10.13039/100010933Xavier Gallart-Palau), grants co-funded by the European Union; the Ministry of Science and Innovation-MCIN, Spain, and the 10.13039/100013101National Research Council/Agencia Estatal de Investigación-10.13039/501100011033AEI, Spain (PID2020-114885RB-C21 to Aida Serra), funded by 10.13039/501100004837MCIN/10.13039/501100011033AEI/10.13039/501100011033. This research was also co-financed by the 10.13039/501100004837Spanish Ministry of Science and Innovation with funds from the 10.13039/501100000780European Union (PRTR-C17.I1); within the framework of the Biotechnology Plan Applied to 10.13039/100018696Health (EVBRAINTARGET-Y7340-ACPPCCOL007 to 10.13039/100010933Xavier Gallart-Palau and Aida Serra) coordinated by the 10.13039/501100014373Institute for Bioengineering of Catalonia (10.13039/501100014373IBEC); the Diputació de Lleida, Spain (PIRS22/03 to 10.13039/100010933Xavier Gallart-Palau and PIRS23/02 to Aida Serra); the Catalan Research Council-10.13039/501100003030AGAUR (2023 LLAV 00056 to 10.13039/100010933Xavier Gallart-Palau and 2022 DI 100 to 10.13039/100010933Xavier Gallart-Palau and Aida Serra). 10.13039/100010933Xavier Gallart-Palau acknowledges a Miguel Servet program tenure-track contract (CP21/00096) from the 10.13039/501100004587ISCIII, awarded in the 2021 call under the 10.13039/100018696Health Strategy Action, co-funded by the 10.13039/501100000780European Union (FSE+). Aida Serra acknowledges a Ramón y Cajal program tenure-track contract (RYC2021-030946-I), funded by 10.13039/501100004837MCIN/10.13039/501100011033AEI/10.13039/501100011033 and by the “10.13039/501100000780European Union NextGenerationEU/PRTR”. Cristina Lorca is supported by a postdoctoral fellowship from the competitive IREP Program of IRBLleida.

## CRediT authorship contribution statement

**Cristina Lorca:** Conceptualization, Investigation, Methodology, Visualization, Writing – original draft, Writing – review & editing. **Aida Serra:** Conceptualization, Investigation, Methodology, Supervision, Writing – review & editing. **Xavier Gallart-Palau:** Conceptualization, Methodology, Supervision, Validation, Writing – review & editing.

## Declaration of competing Interest

None.

## Data Availability

Data will be made available on request.
