# Supplementary material for: Mapping the oxidative stress metabolome in neurology by gas chromatography–mass spectrometry: a systematic review on signature-driven diagnosis and disease monitoring
Source: Redox Biol. 2025 Nov 10;88:103925. doi: 10.1016/j.redox.2025.103925 (PMC12666437; doi:10.1016/j.redox.2025.103925)
Supplement: Multimedia component 1 [file mmc1.docx]

# Supplementary material. **Table 1. Study characteristics and risk of bias assessment.** Quality of studies evaluated according to Newcastle-Ottawa-Scale for cohort studies, QUADAS-2 for diagnostic accuracy study, and Cochrane Risk of Bias tool for randomized trials (RoB 2.0) for randomized control trials.

| **First author, year** | **Metabolites identified** | **Disease and disease type** | **Sample** | **Methodology** | **Study population** | **Type of study** | **Risk of bias tool** | **Selection**  **0-4** | **Comparability 0-2** | **Outcome**  **0-3** | **Total score**  **0-9** |
| --- | --- | --- | --- | --- | --- | --- | --- | --- | --- | --- | --- |
| Andersen, [2019](https://pubmed.ncbi.nlm.nih.gov/30877925/) | Pyroglutamic acid  Acylcarnitine  Phosphatidylcholine (PC)  PC(O-40:5)  PC (O-42:5)  Myo-Inositol | Multiple sclerosis (MS), drug naive  **Neurodegenerative** | Blood serum | Extraction with methanol/chloroform.  N2 drying. Derivatization with methoxyamine in pyridine,  followed by N-Methyl-N-(trimethylsilyl) trifluoroacetamide (MSTFA) with 1% trimethylchlorosilane. | MS cases (n=12): drug naïve for at least three months prior to biospecimen collection, non-Hispanic white, non-smoking males. Controls (n=13): age and body mass index (BMI) frequency matched to cases. | Case-Control | Newcastle-Ottawa Scale | 4 | 2 | 2 | 8 |
| Castillo-Peinado, [2024](https://pubmed.ncbi.nlm.nih.gov/37877569/) | Pyroglutamic acid  Serine  Hydroxybutyric acid  Pyruvic acid | Obstructive sleep apnea (OSA)  **Other** | Sweat | Deproteinisation with methanol (MeOH)–acetonitrile (ACN).  Derivatization: methoximation followed by silylation. | OSA cases (n=41): apnea–hypopnea index (AHI) ≥15 events/h  Controls (n=20): non-OSA subjects AHI <15 events/h.  Exclusion criteria: sample volume <10 μL, age <20 and >70 years, SpO2 of <94%, congestive cardiac failure, hepatic cirrhosis, chronic renal failure, or neuromuscular disease. | Prospective observational case-control | Newcastle-Ottawa Scale | 4 | 1 | 2 | 7 |
| Castor, [2020](https://pubmed.ncbi.nlm.nih.gov/32298384/) | Azelaic acid  Pimelic acid  Suberic acid  Succinic acid | Alzheimer’s disease (AD), pre-symptomatic  **Neurodegenerative** | Urine | Liquid extraction with ethyl acetate. Derivatization: esterification to pentafluorobenzyl (PFB) oximes in the presence of N,N-diisopropylethylamine as a base. | Clinically probable AD (n = 25).  Cognitively healthy (CH) (n = 76), further divided into: asymptomatic low risk individuals (n = 45), and asymptomatic high-risk individuals (n = 31), based on beta amyloid42/tau ratios in the cerebrospinal fluid. | Case-control | Newcastle-Ottawa Scale | 4 | 2 | 2 | 8 |
| [Ceylan, 2018](https://pubmed.ncbi.nlm.nih.gov/29626765/) | Thymine glycol  2,6-Diamino-4-hydroxy-5-N-methylformamidopyrimidine  5-Hydroxy-5-methylhydantoin | Bipolar disorder  **Psychiatric-psychological** | Blood leukocytes | Salting-out DNA extraction from leukocytes, ethanol precipitation for base lesion extraction. Derivatization: trimethylsilylation. | Patients with bipolar disorder (n=32) Healthy controls (n=51), with no medical problems, no personal or first-degree family history of psychiatric or neurological disorders, dementia, mental retardation, cancer, cardiovascular disease, or diabetes.  Diagnoses confirmed via the Structured Clinical Interview for DSM. | Case-control | Newcastle-Ottawa Scale | 4 | 1 | 2 | 7 |
| Dobrzyńska, [2022](https://pubmed.ncbi.nlm.nih.gov/35456059/) | 4-Hydroxy-2-nonenal  Neuroprostanes  Malondialdehyde | Tick-borne encephalitis (TBE)  **Infection** | Blood plasma | Deproteinization with methanol and hexane extraction. Derivatization: oximation with O-(2,3,4,5,6-pentafluorobenzyl) hydroxylamine hydrochloride, followed by silylation with BSTFA + 1% trimethylchlorosilane (TMCS). | TBE patients (n=40) Healthy controls (n=20), without reported infections or clinical symptoms. | Case-control study | Newcastle-Ottawa Scale | 4 | 1 | 2 | 7 |
| Glaab, [2019](https://pubmed.ncbi.nlm.nih.gov/30639291/) | Palmitic acid  Urea | Parkinson’s disease (PD)  **Neurodegenerative** | Blood plasma | Extraction: protein precipitation followed by liquid–liquid extraction into polar and non-polar fractions.  Derivatization: methoximation followed by trimethylsilylation. | PD (n=60) Healthy controls (n=15) age- and gender-matched. | Case-control study | Newcastle-Ottawa Scale | 4 | 2 | 2 | 8 |
| Gomes, [2022](https://link.springer.com/article/10.1007/s12028-021-01285-2) | F2-Isoprostane  Isofurans | Delayed cerebral ischemia (DCI), following aneurysmal subarachnoid hemorrhage (SAH)  **Injury** | Cerebrospinal fluids (CSF) | Vanderbilt GC-MS method for simultaneous quantification of F2-isoprostanes and isofurans: acidified-sample solid-phase extraction (C18 cartridge). Derivatization: PFB esterification followed by TMS silylation | SAH (n = 18)  Controls (n = 6), patients with suspected neurological disease, that resulted normal after testing analysis and imaging. | Prospective observational cohort study | Newcastle-Ottawa Scale | 4 | 1 | 2 | 7 |
| González-Domínguez,  [2015](https://pubmed.ncbi.nlm.nih.gov/25575172/) | Cystine  Pyroglutamic acid  Tryptophan  Aspartic acid  Glutamine  Histidine  Palmitic acid  Oleic acid  Adenosine  Uric acid  2-Oxoglutaric acid  Isocitric acid  Lactic acid  Urea  Glucose | AD, newly diagnosed of sporadic AD, that had not yet received any type of medication  **Neurodegenerative** | Blood serum | Extraction: protein precipitation with methanol/ethanol (1:1), drying under nitrogen. Derivatization: sequential methoximation followed by silylation with MSTFA. | AD (n=23) newly diagnosed, untreated sporadic AD patients.  Healthy controls (n=21), neurologically healthy, age- and sex-matched individuals without significant family history of AD. | Case-control study | Newcastle-Ottawa Scale | 4 | 1 | 2 | 7 |
| Groth, [2023](https://pubmed.ncbi.nlm.nih.gov/37499889/) | 4-Hydroxy-2-nonenal  Malondialdehyde | TBE  **Infection** | CSF | Methanol deproteinization, and hexane liquid-liquid extraction, followed by PFB-oximation and 1% TMCS silylation. | TBE-only patients (n=15) TBE patients with co-infections (n=6)  Control group (n=14), neurologically healthy donors with no meningitis | Case-control study | Newcastle-Ottawa Scale | 4 | 1 | 3 | 8 |
| Jarock, [2020](https://pubmed.ncbi.nlm.nih.gov/32326289/) | 4-Hydroxy-2-nonenal  4-Hydroxy-2-nonenal  4-Oxo-2-nonenal  Malondialdehyde | SAH  **Injury** | Blood | Reactive aldehydes: deproteinization plus hexane Luo’s method. Derivatization: PFB-oximation ± TMS silylation.  Isoprostanes and neuroprostanes: solid-phase extraction after alkaline hydrolysis | SAH patients (n=30) Healthy controls (n=30), age- and sex-matched, with strict exclusion criteria to minimize confounding effects from medication or alcohol use. | Case-control study | Newcastle-Ottawa Scale | 4 | 1 | 2 | 7 |
| Kannenberg, [2017](https://pubmed.ncbi.nlm.nih.gov/26940355/) | Cholestane-3β,5α,6β-triol (C-triol) | Niemann-Pick type C disease  **Neurodegenerative** | Blood plasma | Alkaline saponification followed by liquid–liquid extraction. Derivatization: trimethylsilylation. | Niemann-Pick type C disease patients (n=112) Controls (n=1286) | Diagnostic accuracy study | **QUADAS-2** | Patient selection: low; index test: low; reference standard: low; flow and timing: low. | | | Low risk of bias |
| Konjevod, [2021](https://pubmed.ncbi.nlm.nih.gov/33249139/) | Aminomalonic acid  Malic acid  Fructose | Posttraumatic stress disorder  **Psychiatric-psychological** | Blood plasma | Extraction: deproteinization with cold acetonitrile followed by evaporation.  Derivatization: methoximation with O-methoxyamine, followed by silylation with BSTFA. | Posttraumatic stress disorder (n=102), male veterans. Matched healthy controls (n=102) | Case-control study | Newcastle-Ottawa Scale | 4 | 1 | 2 | 7 |
| Kreilaus, [2016](https://pubmed.ncbi.nlm.nih.gov/26373857/) | 24S-Hydroxycholesterol  7-oxo-cholesterol  7beta-Hydroxycholesterol | Huntington's disease (HD)  **Neurodegenerative** | Post mortem brain tissue | Extraction: alkaline hydrolysis, acidification, solid-phase extraction, and elution with hexane/MTBE.  Derivatization: silylation with BSTFA + 1% TMCS. | HD (n=13) Controls (n=13), mean age matched, brain tissue neuropathologically screened and confirmed free of degenerative pathologies. | Case-control study | Newcastle-Ottawa Scale | 4 | 2 | 3 | 9 |
| Mussap, [2020](https://pubmed.ncbi.nlm.nih.gov/33238400/) | Indoleacetic acid  Aminomalonic acid  Cysteine  Tryptophan  Uric acid  Quinic acid  Myo-Inositol | Autism  **Psychiatric-psychological** | Urine | Derivatization: methoximation with O-methoxyamine hydrochloride followed by silylation with BSTFA. | Autistic children (n=31, 23 males, 8 females), 2 to 11 years, diagnosed per DMS-5. Control (n=26, 16 males, 10 females), neurotypical, age and ethnicity matched.  **Pediatric** | Case-control study | Newcastle-Ottawa Scale | 4 | 2 | 2 | 8 |
| Piras, [2022](https://pubmed.ncbi.nlm.nih.gov/36535959/) | Aspartic acid  Serine  Pyruvate  Urea | Fibromyalgia  with self-reported electromagnetic sensitivity  **Other** | Blood plasma | Extraction: modified Folch method (methanol/chloroform/water).  Derivatization: methoximation with methoxyamine hydrochloride in pyridine, followed by silylation with MSTFA. | Fibromyalgia and electromagnetic sensitivity (n=31, 21 females, 2 males) Healthy controls (n=23, 21 females, 2 males) | Case-control study | Newcastle-Ottawa Scale | 3 | 1 | 2 | 6 |
| Poddighe, [2017](https://pubmed.ncbi.nlm.nih.gov/28720279/) | Glutamate  Ornithine  Pyroglutamic acid  Glutamine  Threonic acid  Phosphoric acid  Myo-Inositol  Fructose | MS  **Neurodegenerative** | Blood plasma | Extraction: methanol/chloroform (1:1) with water (modified biphasic method).  Derivatization: methoximation with methoxyamine hydrochloride in pyridine, followed by silylation with MSTFA. | MS patients (n=32) Healthy controls (n=33), age- and ethnicity-matched. | Case-control study | Newcastle-Ottawa Scale | 4 | 1 | 2 | 7 |
| Signorini, [2018](https://pubmed.ncbi.nlm.nih.gov/29233794/) | Neuroprostane 10-F  Neuroprostane 4-F  Neuroprostane 10  Neuroprostane 4 | MS  Autism  Rett syndrome  Down syndrome  **Neurodegenerative**  **Psychiatric-psychological Other** | Blood plasma | Extraction: sequential solid-phase extraction using C18 and NH₂ cartridges after plasma acidification.  Elution with ethyl acetate:methanol:acetic acid (10:85:5).  Derivatization: formation of pentafluorobenzyl esters, followed by silylation with BSTFA + DIPEA. | Relapsing-remitting MS patients (n = 16)  Rett syndrome patients (n = 20, all females with MECP2 mutations)  Autistic patients (n = 9, 7 males, 2 females), 13.5 ± 4.6 years  Down syndrome patients (n = 16), 24.9 ± 4.7 years  Healthy controls (n = 61), age- and gender-matched.  **Pediatric autistic patients** | Multi-arm case-control study. | Newcastle-Ottawa Scale | 3 | 2 | 2 | 7 |
| [Sinningen, 2023](https://pubmed.ncbi.nlm.nih.gov/37392928/) | Nitrate  Nitrite | Attention deficit hyperactivity disorder (ADHD)  **Psychiatric-psychological** | Blood plasma | Derivatization: PFB-ester formation (60 min at 50 °C); acetone evaporated under nitrogen. Extraction of reaction products with ethyl acetate, dried over anhydrous Na₂SO₄. | Adult ADHD patients (n = 29),  Healthy controls (n = 32).  Exclusion criteria: other psychiatric disorders, substance abuse, medication use, neurological or serious internal diseases, and IQ < 85. | Prospective case-control study | Newcastle-Ottawa Scale | 4 | 1 | 2 | 7 |
| Su, [2022](https://pubmed.ncbi.nlm.nih.gov/34990999/) | Aspartic acid  Glutamine  Homocysteine  Methionine  Cysteine  Ornithine  Linoleic acid  Guanosine  Hypoxanthine  Xanthine  Fumaric acid  Pyruvic acid  Myo-Inositol | Methamphetamine dependence  **Psychiatric-psychological** | Peripheral blood mononuclear cells (PBMCs) | Extraction: proteins precipitated with cold methanol/chloroform (3:1).  Derivatization: methoximation with methoxyamine in pyridine, followed by silylation with MSTFA + 1% TMCS. | Methamphetamine use disorder patients (n=40), all males meeting DSM-5 criteria for severe use.  Healthy controls (n=38), no history of drug use, psychiatric, neurological, or major physical disorders. | Case-control study | Newcastle-Ottawa Scale | 4 | 1 | 2 | 7 |
| Trezzi, [2017](https://pubmed.ncbi.nlm.nih.gov/28843022/) | Threonic acid  Dehydroascorbic acid  Fructose | PD, early  **Neurodegenerative** | CSF | Extraction: CSF mixed with methanol/water (8:1).  Derivatization: methoximation with methoxyamine hydrochloride in pyridine, followed by silylation with MSTFA. | Sporadic PD patients (n = 44), early stage. Healthy controls (n = 43), matched by age, sex, and education, recruited from spouses and community volunteers without neurological disease. | Case-control study (nested in prospective cohort) | Newcastle-Ottawa Scale | 4 | 2 | 2 | 8 |
| Wang, [2017](https://pubmed.ncbi.nlm.nih.gov/28111353/) | Glutamate  Glycine  Serine  Tryptophan  Hypoxanthine  Purine  Citrate  Pyruvic acid  Urea | Acute ischemic stroke  **Injury** | Blood serum | Extraction: methanol/water (8:1).  Derivatization: Methoximation with methoxyamine hydrochloride in pyridine, followed by silylation with MTBSFA + 1% TMCS. | Acute ischemic stroke patients (n = 40; 24 anterior, 12 posterior, 3 both, 1 lacunar infarction) Healthy controls (n = 29), age- and sex-matched  Patients recruited within 7 days of symptom onset; diagnosis confirmed by MRI and clinical evaluation per AHA/ASA guidelines.  Exclusion: renal/liver disease, malignancy, autoimmune disease, hypothyroidism. | Case-control study | Newcastle-Ottawa Scale | 4 | 1 | 2 | 7 |
| Yen, [2015](https://pubmed.ncbi.nlm.nih.gov/26271312/) | F2-Isoprostanes  F4-neuroprostane | Traumatic brain injury (TBI)  **Injury** | Blood plasma | Derivatization: conversion to pentafluorobenzyl ester and trimethylsilyl ether derivatives. | TBI patients (n = 15), moderate to severe  Healthy controls (n = 11).  CSF and plasma collected 6–10 days post-surgery in TBI patients and once in controls. | Prospective observational cohort study | Newcastle-Ottawa Scale | 4 | 1 | 2 | **7** |
| Zarrouk, [2020](https://pubmed.ncbi.nlm.nih.gov/33272182/) | 24S-Hydroxycholesterol  25-Hydroxycholesterol  7beta-Hydroxycholesterol | AD  **Neurodegenerative** | Blood plasma | Extraction: organic solvent-based lipid extraction.  Derivatization: conversion of sterols to trimethylsilyl ether derivatives. | AD patients (n=56)  Healthy controls (n=97) | Case-control study | Newcastle-Ottawa Scale | 3 | 1 | 2 | 6 |
| Zhao, [2022](https://pubmed.ncbi.nlm.nih.gov/35206569/) | Elaidic acid  Inosine  Uric acid  3,4-Dihydroxybenzeneacetic acid  Epinephrine  6-Phosphogluconic acid | Chronic fatigue symptoms in adolescents  **Other** | Urine | Extraction: methanol extraction after urease treatment.  Derivatization: methoximation with methoxyamine hydrochloride in pyridine, followed by silylation with BSTFA (1% TMCS). | Chronic fatigue syndrome students (n=46), all males, 15–18 years; subdivided into exercise intervention (n=23) and non-intervention (n=23)  Healthy controls (n=24) males, age- and sex-matched  **Pediatric/adolescent** | Randomized control trial | Cochrane Risk of Bias tool for randomized trials (RoB 2.0) | Randomization process: low; deviations from intended interventions: some concerns; missing outcome data: low; outcome measurement: some concerns; selection of the reported results: low. | | | Low risk/some concerns regarding risk of bias |
